# Population genetic structure and evolution of Batesian mimicry in *Papilio polytes* from the Ryukyu Islands, Japan, analyzed by genotyping‐by‐sequencing

**DOI:** 10.1002/ece3.7092

**Published:** 2020-12-24

**Authors:** Yukuto Sato, Kaori Tsurui‐Sato, Mitsuho Katoh, Ryosuke Kimura, Haruki Tatsuta, Kazuki Tsuji

**Affiliations:** ^1^ Center for Strategic Research Project University of the Ryukyus Okinawa Japan; ^2^ Department of Agro‐Environmental Sciences Faculty of Agriculture University of the Ryukyus Okinawa Japan; ^3^ The United Graduate School of Agricultural Sciences Kagoshima University Kagoshima Japan; ^4^ Department of Human Biology and Anatomy Graduate School of Medicine University of the Ryukyus Okinawa Japan

**Keywords:** female‐limited mimetic polymorphism, MIG‐seq, molecular phylogeny, population genetics, SNP analysis

## Abstract

Batesian mimicry is a striking example of Darwinian evolution, in which a mimetic species resembles toxic or unpalatable model species, thereby receiving protection from predators. In some species exhibiting Batesian mimicry, nonmimetic individuals coexist as polymorphism in the same population despite the benefits of mimicry. In a previous study, we proposed that the abundance of mimics is limited by that of the models, leading to polymorphic Batesian mimicry in the swallowtail butterfly, *Papilio polytes,* on the Ryukyu Islands in Japan. We found that their mimic ratios (MRs), which varied among the Islands, were explained by the model abundance of each habitat, rather than isolation by distance or phylogenetic constraint based on the mitochondrial DNA (mtDNA) analysis. In the present study, this possibility was reexamined based on hundreds of nuclear single nucleotide polymorphisms (SNPs) of 93 *P. polytes* individuals from five Islands of the Ryukyus. We found that the population genetic and phylogenetic structures of *P. polytes* largely corresponded to the geographic arrangement of the habitat Islands, and the genetic distances among island populations show significant correlation with the geographic distances, which was not evident by the mtDNA‐based analysis. A partial Mantel test controlling for the present SNP‐based genetic distances revealed that the MRs of *P. polytes* were strongly correlated with the model abundance of each island, implying that negative frequency‐dependent selection interacting with model species shaped and maintained the mimetic polymorphism. Taken together, our results support the possibility that predation pressure, not isolation by distance or other neutral factors, is a major driving force of evolution of the Batesian mimicry in *P. polytes* from the Ryukyus.

## Graphical abstract

Batesian mimicry is a striking example of Darwinian evolution, receiving protection from predators. We found that the swallowtail butterfly *Papilio polytes*, which shows polymorphic Batesian mimicry, has the mimic ratios explained by the model species abundance of each habitat, rather than isolation by distance or phylogenetic constraint in the Ryukyu Islands, Japan. Our results support the possibility that predation pressure, not neutral factors, is a major driving force of evolution of the Batesian mimicry in *P. polytes* populations of the Ryukyus.

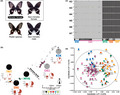

## 1 INTRODUCTION

Batesian mimicry is an often mentioned and striking example of Darwinian evolution (Bates, 1862; Cott, 1940; Edmunds, 1974; Kunte, 2009; Rettenmeyer, 1970; Ruxton et al., 2018). In this mimicry system, prey species resemble harmful or unpalatable model species, receiving protection from predators through visual imitation of toxic or otherwise defensive models. In some butterfly species, however, Batesian mimicry is found only in a subset of individuals of the population (Kunte, 2009; Mallet & Joron, 1999; Wallace, 1865; Wickler, 1968). Such polymorphism in Batesian mimicry has long been a matter of debate (Kunte, 2009; Mallet & Joron, 1999) as it is counterintuitive to the benefits of mimicry for avoiding predation (Kunte, 2009; Mallet & Joron, 1999). Functional variation of the autosomal gene *doublesex* (*dsx*) is related to the polymorphism of Batesian mimicry in butterflies of the genus *Papilio* (Clarke & Sheppard, 1972; Iijima et al., 2018, 2019; Komata et al., 2016; Kunte et al., 2014; Nishikawa et al., 2015; Palmer & Kronforst, 2020; Zhang et al., 2017). However, determining the ecological factors and evolutionary mechanisms that shape and maintain mimetic polymorphism is a long‐standing unresolved challenge.

In a previous study, we proposed that negative frequency‐dependent selection (NFDS) interacting with model species explains the polymorphic Batesian mimicry of the swallowtail butterfly *Papilio polytes* L. from the Ryukyu Islands in Japan (Tsurui‐Sato et al., 2019). The FDS model (Barrett, 1976; Kunte, 2009; Turner, 1978) predicts that the number of mimics in the population depends on the abundance of the model species in the habitat, because the defensive benefit of mimicry increases when the toxic or unpalatable model is more abundant. But if the mimics become overabundant relative to the models, the advantage of the mimicry decreases (Barrett, 1976). Accordingly, the mimic ratio (MR; proportion of mimics in the population) is expected to approach an equilibrium with equal fitness between mimetic and nonmimetic types (Kunte, 2009), negatively determined by an abundance of model species as an ecological factor.

**Figure 1:**
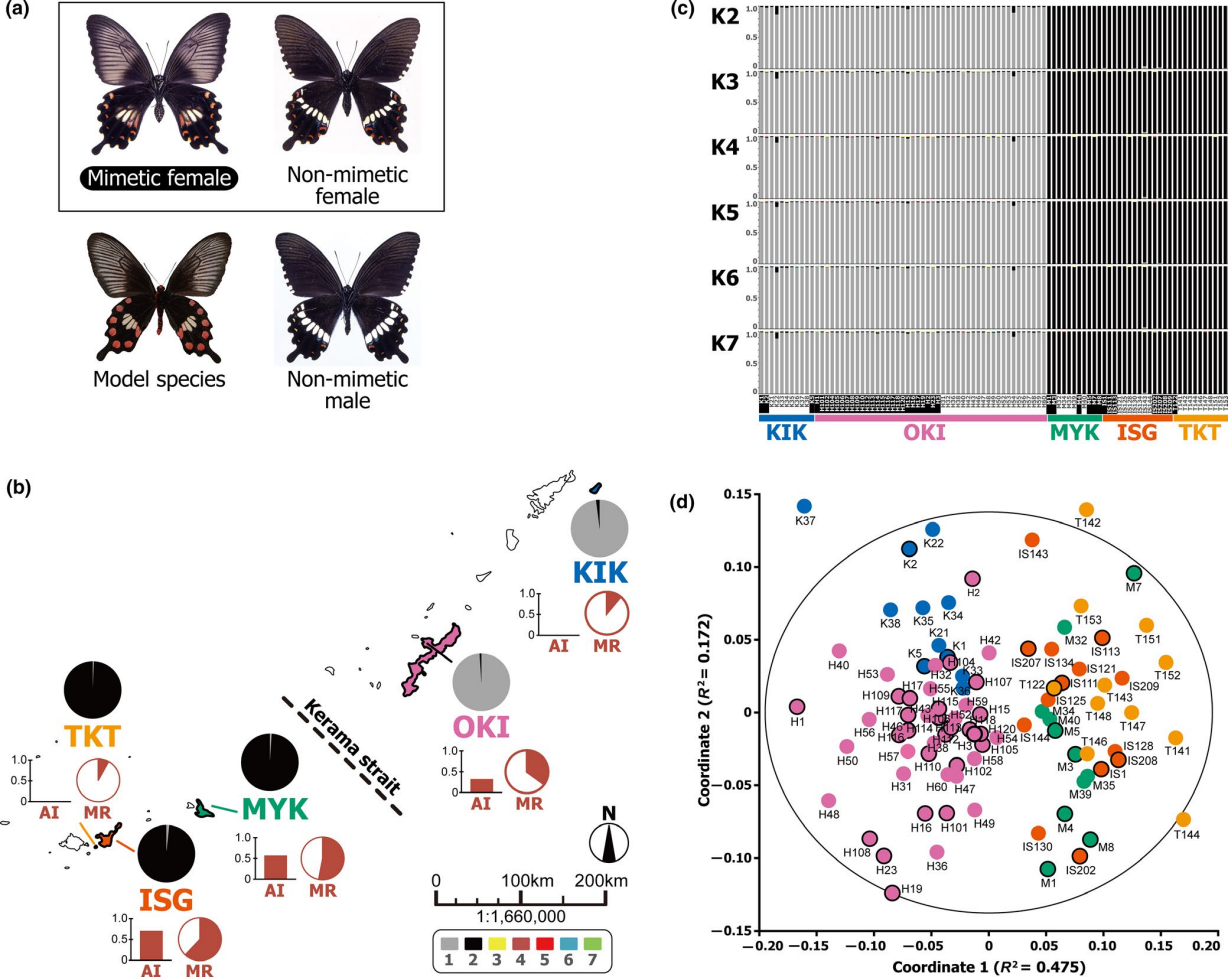
Mimicry system, sampling sites, and estimated population genetic structure of *Papilio polytes*. (a) Mimetic females of *P. polytes* resemble the model species *Pachliopta aristolochiae*, while nonmimetic females are similar in appearance to males. Butterfly photographs were taken by Senshi Nobayashi. (b) Map of the five islands where sampling was performed in this study (denoted by thick outline): KIK, Kikai; OKI, Okinawa; MYK, Miyako; ISG, Ishigaki; TKT, Taketomi. Pie charts above the island names show the averaged population composition assigned to individuals of the island at *K* (number of populations) equaling 2 in the population assignment tests shown in panel (c). Orange colored bar and pie charts below the names of the island show advantage index (AI) of Batesian mimicry and mimic ratio (MR) of the island presented in Tsurui‐Sato et al. (2019) and analyzed in this study. (c) Individual‐level population compositions estimated by the population assignment tests at *K* equaling 2–7. Colors within the bar chart indicate respective populations 1–7 as denoted in panel (b). Mimetic individuals are denoted as white outlined letters in the sample names. (d) Coordinates of two‐dimensional nMDS (nonmetric multidimensional scaling) of the SNP profiles. K1–K38, H1–H120, M3–M40, IS1–IS209, and T121–T153 are the samples from the Islands KIK, OKI, MYK, ISG, and TKT, respectively. Horizontal and vertical axes correspond to the estimated two‐dimensional coordinates 1 and 2 where ranked differences in similarity scores among individuals were preserved. The normalized stress value of this plot was 0.320. Mimetic individuals are outlined in black

This NFDS model for the maintenance of Batesian mimicry polymorphism may be evaluated by focusing on *P. polytes* from the Ryukyu Islands as a model system (Tsurui‐Sato et al., 2019). This species is a common swallowtail butterfly in Southeast Asia and the southern part of East Asia, and exhibits polymorphic Batesian mimicry in females (Clarke & Sheppard, 1972; Ford 1975; Kunte et al., 2014; Katoh et al., 2017). In the Ryukyu Islands, some females mimic unpalatable *Pachliopta aristolochiae* as a defense against avian predators (Katoh et al., 2017; Uesugi, 2000), while others resemble males (Figure 1a). Interestingly, the MRs vary among the Ryukyu Island populations and tend to be high where more of the model *P. aristolochiae* are present (Tsurui‐Sato et al., 2019; Uesugi, 2000). In particular, after the model species immigrated to Miyako Island (MYK in Figure 1b) and became established, the MR of this island increased rapidly from 1975 to 1989 (Uesugi, 2000), implying local adaptation through NFDS (Tsurui‐Sato et al., 2019).

As an alternative hypothesis, polymorphic Batesian mimicry may be explained by sexual selection and ecological–physiological trade‐offs (Burns, 1966; Cook et al., 1994; Katoh et al., 2020; Ohsaki, 2005; Vane‐Wright, 1984), or neutral evolutionary processes such as isolation by distance and phylogenetic constraint. In the simple neutral process, the mimetic and nonmimetic phenotypes have similar fitness independent of their frequencies. Accordingly, the MRs in local populations change through genetic drift and migration (Ackermann & Cheverud, 2004; Wright, 1943). Given this scenario, geographically closer populations (i.e., neighboring island populations), which are expected to be genetically closer to each other, should exhibit similar MRs irrespective of the quantity of mimetic models on each island.

Our previous study (Tsurui‐Sato et al., 2019) examined the possible association between the MR of *P. polytes* and the abundance of the mimetic model in the Ryukyu Islands, with respect to the NFDS hypothesis. The alternative, isolation‐by‐distance view of the MR variation among islands was tested by genetic analyses based on mitochondrial DNA (mtDNA). Strong correlation of the MRs of *P. polytes* with mimetic model abundance was found in five islands of the Ryukyu, rather than with geographic or genetic distances, incorporating newly added fieldwork data (Tsurui‐Sato et al., 2019). In particular, the northern island populations were thought to be relatively recent immigrants having experienced population expansion, while their MRs correlated with the local abundance of model species that was probably caused by rapid evolution through NFDS (Tsurui‐Sato et al., 2019). These results, however, may have suffered from incomplete lineage sorting or other biases because they are primarily based on a single locus. Hence, to evaluate the insights gathered from our former study, we address the population genetic characteristics and mimetic evolution of *P. polytes* of the same Ryukyu Islands, based on a much larger set of single nucleotide polymorphisms (SNPs) of nuclear genomes that enables more averaged and comprehensive evaluation of genetic diversity of the populations.

## 2 MATERIALS AND METHODS

### 2.1 Sample collection and DNA extraction

Sample *P. polytes* were collected as described in our previous study (Tsurui‐Sato et al., 2019) from eight islands of the Ryukyu Archipelago (the Ryukyus), southern Japan (Kikai, Amami, Okinawa, Aguni, Miyako, Tarama, Ishigaki, and Taketomi Islands). Among these samples, we analyzed 95 females from the following five islands, where more than ten individuals of mimetic and nonmimetic females were obtained: 11, 46, 12, 14, and 12 individuals of Kikai (abbreviated as KIK), Okinawa (OKI), Miyako (MYK), Ishigaki (ISG), and Taketomi (TKT), respectively (Figure 1). These are the same samples analyzed for mtDNA in the study of Tsurui‐Sato et al. (2019), apart from an additional four and two samples from MYK and ISG, respectively, which achieved successful PCR amplification and were included in this study for nuclear SNP analysis. Butterflies were caught using a bug‐net between 2014 and 2016, examined to record the sex and mimic type, and stored at −30°C until DNA extraction. DNA was extracted from the entire middle and hindlegs of the frozen samples using the DNeasy Blood and Tissue Kit (Qiagen) after hand shearing, manual homogenization, and enzymatic digestion of the samples by proteinase K, as described by Tsurui‐Sato et al. (2019). The eluted DNA was quantified using a NanoDrop 2000C spectrophotometer (Thermo Scientific) and stored at −30°C.

### 2.2 PCR amplification and genotyping by sequencing for nuclear SNP analysis

To obtain genotyping data for random SNPs from the nuclear genome using a PCR‐based method, we applied multiplexed intersimple sequence repeat (ISSR) genotyping by sequencing (MIG‐seq) analysis (Suyama & Matsuki, 2015). Since frozen leg samples of *P. polytes* butterflies are relatively small in size and weight, the total genomic DNA obtained from these samples was relatively low and the DNA quality was variable due to diverse field and sample storage conditions (Table S1; 15.44 ± 0.69 ng/μl (mean ± *SE*) ranging from 1.6 to 44.3; 2.03 ± 0.08 of OD_260_/OD_280_ ranging from 0.54 to 6.50). Therefore, it was difficult to apply SNP analysis methods such as restriction site‐associated DNA sequencing (RAD‐seq) that require steady large amounts of high‐quality DNA to our *P. polytes* samples. On the other hand, PCR‐based methods, such as MIG‐seq, are capable of analyzing smaller amounts of DNA, although the number of consensus SNPs identified among individuals may be limited compared to that with RAD‐seq or whole‐genome resequencing methods (Davey et al., 2011; Suyama & Matsuki, 2015).

To amplify and sequence the flanking regions of ISSRs from genomic DNA using ISSR‐targeted PCR primers of the MIG‐seq method, two rounds of PCR were performed as described by Suyama and Matsuki (2015). The reaction mixture of the 1st round PCR included 1.0 μl of sample DNA, 0.2 μM of each MIG‐seq set‐1 primer (Suyama & Matsuki, 2015), 0.035 μl of Multiplex PCR Enzyme Mix, and 3.5 μl of 2 × Multiplex PCR Buffer from the Multiplex PCR Assay Kit Ver. 2 (Takara Bio) for each sample. The thermal cycling profile of the 1st round PCR was as follows: 94°C for 1 min followed by 27 cycles at 94°C for 30 s, 48°C for 1 min, and 72°C for 1 min, with a final extension at 72°C for 10 min.

The 1st‐round PCR product was diluted 50‐fold in RNase‐free water (Thermo Fisher Scientific/Invitrogen) and subjected to 2nd‐round indexing PCR to add the dual‐index tags (A5 and A7 series) and flow cell binding site sequences of the Illumina DNA sequencer platforms (Illumina). The PCR mixture contained 2.5 μl of diluted 1st‐round PCR product, 0.2 μM of each forward and reverse indexing primer, 0.24 μl of PrimeSTAR GXL DNA Polymerase (Takara Bio), 0.96 μl of dNTP mixture, and 2.4 μl of 5 × PrimeSTAR GXL Buffer (Takara Bio). The thermal cycling profile was as follows: 94°C for 1 min followed by 15 cycles at 98°C for 10 s, 54°C for 15 s, and 68°C for 30 s. The amplified MIG‐seq DNA libraries were equivalently pooled among samples, extracted from 1.0% L03 agarose gel (Takara; sizes ranging from ca. 400 to 800 bp) using a MinElute Gel Extraction Kit (Qiagen), purified using AMPure XP magnetic beads (Beckman Coulter) using a standard purification protocol, quantified by a Qubit 3.0 fluorometer (Thermo Fisher Scientific) and Agilent 2100 Bioanalyzer (Agilent), and sequenced in a single lane of the HiSeq X platform (Illumina) to generate 2 × 151‐bp paired‐end sequences.

### 2.3 Quality‐based sequence data filtering and *de novo* SNP calling

Raw sequencing data of MIG‐seq amplicons from the genomic DNA of *P. polytes* (DRA accession number: DRA010473) underwent quality filtering using relevant software/scripts and *de novo* SNP calling using the software package Stacks version 1.45 (Catchen et al., 2013). The first 17 nucleotides of the 5′‐ends of each read were uniformly deleted to remove MIG‐seq forward primer sequences using Cutadapt version 1.14 (Martin, 2011). The adapter primer‐derived sequences that sometimes remained or were repeated in the opposite 3′‐ends of each read were carefully removed by Cutadapt, twice, using a ≥ 8‐bp length accordance and a ≤ 10% base mismatch. Finally, the low‐quality (Phred score < 10) 3′‐tails of each read were trimmed by Cutadapt and the shorter sequences (<120 bp in length) were excluded using a custom Perl script.

**Figure 2:**
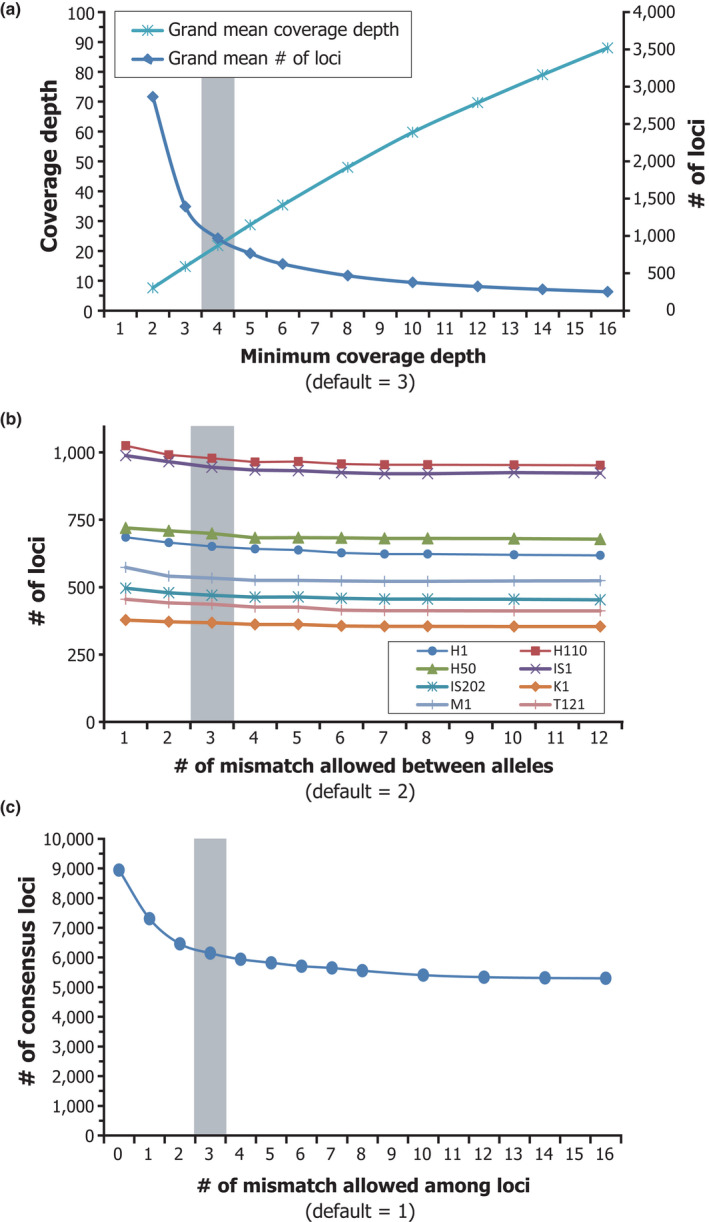
Examination of parameter settings of *de novo* SNP calling analysis using the Stacks software. These analyses were performed using preliminary sequence data of eight *P. polytes* individuals from all five islands with an average of 129,999 ± 5,245 (*SE*) of quality‐filtered forward reads per sample. (a) Relationship between the values of minimum coverage depth and the obtained number of SNP loci and their grand mean depth across loci and individuals. Grand mean depth increased and obtained number of loci decreased when the threshold values of minimum coverage depth became higher. (b) Relationship between the maximum number of mismatches (in base pairs) between alleles of the same individual and the number of obtained SNP loci. When the threshold of mismatches allowed between alleles increased, the number of obtained loci decreased (overmerged), and vice versa. (c) Relationship between the maximum number of mismatches (in base pairs) among alleles from different individuals and the number of consensus SNP loci obtained. When the threshold of mismatches allowed among alleles increased, the number of consensus loci decreased (overmerged), and vice versa

The quality‐filtered forward reads (read R1) were analyzed for *de novo* SNP calling. First, all forward reads from each sample were clustered into putative loci within an individual based on sequence similarity using the program Ustacks that is provided in the software package Stacks. Threshold values of minimum coverage depth of respective loci and maximum number of mismatched bases among alleles were set to 4 and 3 (2.2%–2.5% per locus), respectively, based on preliminary analysis using eight individuals from all five islands (Figure 2a,b). Next, consensus loci among individuals were identified using the program Cstacks, with a threshold value of 3 for the maximum number of mismatch bases among alleles from other individuals (2.2%–2.5% per locus), as determined based on preliminary analysis of the same individuals as above (Figure 2c). Finally, SNP calling was performed using the programs Sstacks and Populations to generate an SNP matrix table with less than 50% missing values for each locus across all individuals. All individuals were treated provisionally as one population (predefinition of subpopulations was not applied) to avoid biased output of missing values affected by a given subpopulation structure. One SNP site was randomly selected from each locus for outputting SNP genotypes using the option “write‐random‐snp” for the Populations.

### 2.4 Population genetic and molecular phylogenetic analyses

To examine the phylogeographic and evolutionary history of *P. polytes* from the Ryukyu Islands in relation to their Batesian mimicry, we performed population genetic and molecular phylogenetic analyses. Among the generated SNP data matrix, we focused on loci with less than 20% missing values across all 95 individuals. We removed the individuals having more than 30% missing values across those loci, which was probably due to the relatively low quality of initial genomic DNA. Then, the SNP loci with less than 20% of recalculated missing values across the remaining individuals were used for the following genetic analysis.

The chi‐squared tests were performed to remove the SNP loci indicating significant deviation from the Hardy–Weinberg equilibrium (HWE; *p* < .05) in any one of the five island populations, using Arlequin version 3.5.2.2 (Excoffier & Lischer, 2010). On the basis of the remaining multilocus SNP genotypes, the population genetic structure was estimated using the program Structure, version 2.3.4 (Pritchard et al., 2000). The implemented admixture model was applied to cluster individuals into genetically similar subpopulations considering each SNP locus as genetically independent. The length of burn‐in period and number of Markov chain Monte Carlo (MCMC) iterations after burn‐in were set to 50,000. Population genetic parameters including observed and expected heterozygosity, pairwise *F*_ST_ among island populations (Slatkin, 1995) standardized by the estimated divergence times, and average gene diversity (or average heterozygosity; Nei, 1987) over SNP sites were calculated using Arlequin.

SNP‐based phylogenetic analysis was performed with both concatenation‐ and coalescent mode‐based methods. First, multilocus SNP genotype data were concatenated in a random order of alleles for each locus using a custom Perl script, since the MIG‐seq loci were considered to be independent, and thus, the gametic phases were unknown. The obtained multi‐FASTA formatted data were analyzed to estimate the molecular phylogenetic network tree based on the NeighborNet algorithm (Bryant & Moulton, 2004) and Kimura's two‐parameter model of nucleotide substitution (Kimura, 1980) using the SplitsTree 5 version 5.1.7 (Huson & Bryant, 2006). Coalescent model‐based phylogenetic analysis was performed using the software package SNAPP (SNP and AFLP Package for Phylogenetic analysis; Bryant et al., 2012) implemented in BEAST 2 version 2.6.2 (Bouckaert et al., 2014, 2019). The analysis was performed with default parameters and settings including the Jukes–Cantor model (Jukes & Cantor, 1969) of nucleotide substitution and 10,000,000 MCMC iterations.

Based on the same concatenated SNP site data, a molecular phylogenetic tree was estimated by the neighbor‐joining (NJ) method (Saitou & Nei, 1987) based on Kimura's two‐parameter model of nucleotide substitution (Kimura, 1980) using MEGA 7 version 7.0.14 (Kumar et al., 2016). The average number of pairwise differences and Nei's net number of nucleotide differences *D_A_* (Nei & Li, 1979) of SNP sites among the five island populations was also estimated. Based on the genetic distance *D_A_* of the SNP sites, the population‐level tree among the islands was inferred by the NJ method. Genetic differentiation (*Φ*_ST_) of the SNP sites among the islands was also calculated using *D_A_* divided by the average number of pairwise differences among islands. Coordination of two‐dimensional nonmetric multidimensional scaling (nMDS) of the SNP profile was estimated using PAST version 4.03 (Hammer et al., 2001) based on the Kimura's two‐parameter model of nucleotide substitution (Kimura, 1980), to calculate similarity scores among the samples.

### 2.5 Mimic ratio and advantage index of Batesian mimicry

The MR and the advantage index (AI) of Batesian mimicry of *P. polytes* have been calculated as described in Tsurui‐Sato et al. (2019) as follows:

$$\mathrm{MR}=\frac{\mathrm{mimic}\;P.polytes\;\mathrm{females}}{\mathrm{nonmimic}\;P.polytes\;\mathrm{females}+\mathrm{mimic}\;P.polytes\;\mathrm{females}}$$

$$\mathrm{AI}=\frac{P.aristolochiae}{\mathrm{mimic}\;P.polytes\;\mathrm{females}+P.aristolochiae}$$

The definition of MR is same with the relative abundance of mimics defined by Sekimura et al. (2014). AI provides the degree of advantage for mimic *P. polytes* and predicts that the MR equilibrium corresponds to the local abundance of model species according to the NFDS hypothesis. AI value increases with the number of model species relative to that of mimic *P. polytes* and the higher AI is expected to confer the higher MR. The present definition of AI is modified from those of Uesugi (2000) and Sekimura et al. (2014) by removing “nonmimic *P. polytes* females” from the denominator because the nonmimic *P. polytes* do not possess warning signals for avian predators and consequently be not thought to affect the mimicry advantage.

### 2.6 Mantel and partial Mantel tests regarding the evolution of Batesian mimicry

Possible associations among genetic distance *D_A_*, geographic distance, and MR of the five island populations of *P. polytes* were assessed by the Mantel test (Mantel, 1967) and partial Mantel test (Smouse et al., 1986). The *D_A_*s of the SNP sites among the five island populations, estimated in the previous section, were used as average genetic distances between islands. Geographic distances among the islands were measured by Tsurui‐Sato et al. (2019), and the same data were used in the present analysis. These were distances in kilometers between the centers of sampling regions (i.e., city hall or downtown area) of each island (Figure 1a). However, the location of the Nakijin village office was used for OKI because the butterflies were collected in the Nakijin region, in the northern part of OKI. The MR and the advantage index (AI) of Batesian mimicry of *P. polytes* used in this study were calculated by Tsurui‐Sato et al. (2019) based on the methods of Uesugi (2000) and Sekimura et al. (2014). The Mantel test was performed using Arlequin, and the partial Mantel test was performed using a custom R script developed on the R platform version 3.4.2 (R Core Team, 2017), based on 10,000 permutation tests according to Method 1 by Legendre (2000).

## 3 RESULTS

### 3.1 General results of genotyping by sequencing

We obtained a total of 115,550,624 pairs of raw DNA sequences with an average of 1,216,322 ± 51,480 (mean ± *SE*) per sample. Following the base call quality‐ and sequence length‐based filtering of the forward reads (see Materials and Methods), a total of 94,897,949 reads (998,926 ± 44,136 per sample) with overall mean Phred quality score 30.54 ± 0.02 per base position (ranging from 20.3 to 36.7) and ≥120 bp in length remained. Based on these quality‐filtered forward reads, on average, 4,864 ± 164 SNP loci were obtained for each sample with a mean coverage depth of 48 ± 0.24. Among the 95 individuals, 1,258, 799, 517, 233, and 82 consensus SNP loci were identified with less than 50, 40, 30, 20, and 10 percentage missing values for each locus, respectively. Focusing on the 233 loci with <20% missing rates, two individuals (M9 of MYK and T121 of TKT) showed >30% missing values per sample and were excluded from the analysis. These two samples, however, did not exhibit remarkably low concentration or quality of total DNA (18.2 ng/μl and 2.21 of OD_260_/OD_280_ in M9; 15.5 ng/μl and 2.23 of OD_260_/OD_280_ in T121). Based on the recalculated missing rates, a total of 259 SNP loci with <20% missing values for each locus from 93 samples were used in the population genetic and phylogenetic analyses.

### 3.2 Population genetic structure and characteristics of *P. polytes* in the Ryukyu Islands

**Figure 3:**
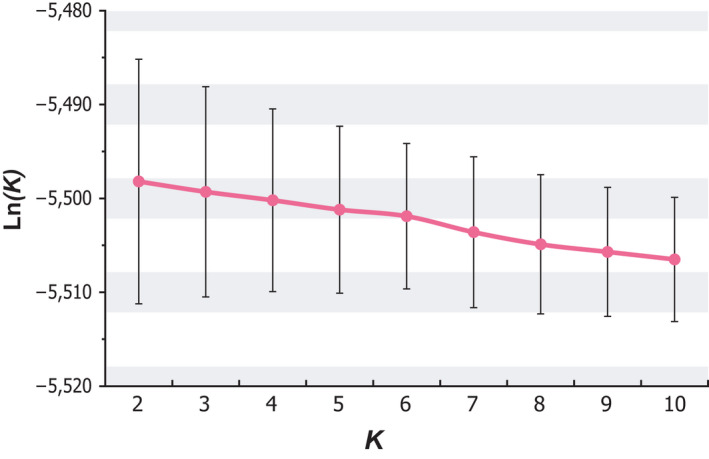
Log‐likelihood scores and standard errors in population assignment tests shown in Figure 1c at *K* equaling 2–10. Vertical and horizontal axes indicate log‐likelihood score and predefined number of population *K*, respectively. Magenta points and line show mean values of log‐likelihood score at each *K*, and vertical bars exhibit their standard errors

The SNP‐based population genetic analyses indicated that two almost distinct *P. polytes* populations exist in the five Ryukyu Islands (Figure 1). The analysis was performed using 238 SNP site data from the 93 individuals after excluding 21 sites that showed significant deviation from the HWE at *p* < .05. The results of the population assignment test showed that one population (indicated by gray in Figure 1b,c) corresponded to the northern islands (KIK and OKI), and another (indicated by black in Figure 1b,c) to the southern islands (MYK, ISG, and TKT). This assignment test showed highest mean log‐likelihood value when the number of population (*K*) was set to two (−5,498.2; Figure 3); however, these values were not remarkably differed if the *K* was increased to seven or even 10 (5.4–8.3 decrements, respectively).

The individual‐level plot of nMDS exhibited a consistent pattern, in which the northern and southern island populations clustered separately (Figure 1d). The northern individuals (colored in blue and pink in Figure 1d) were plotted in lower value spaces of coordinate 1 (horizontal axis of the plot) and the southern ones (colored in green, orange, and yellow in Figure 1d) in higher value spaces. The estimated population genetic indicators based on these SNP sites (not a whole genomic average) showed relatively lower average gene diversity over SNP sites in the northern island populations (0.035–0.048), while those of the southern islands were relatively higher (0.057–0.102) (Table 1). In particular, the southern ISG and TKT had larger numbers of polymorphic sites (23 and 29) relative to their smaller sample sizes.

**Table 1:** Population genetic statistics of SNP loci/sites used in the analysis of the present study

Island	No. of individuals	# loci	No. of usable loci (<5.0% missing)	No. of polymorphic sites	No. of sites with private alleles	*H*_O_ (±*SD*)^a^	*H*_E_ (±*SD*)^b^	Average gene diversity over usable loci (±*SD*)^c^
KIK	11	238	56	14	2	0.234 (±0.2161)	0.194 (±0.1363)	0.048 (±0.0298)
OKI	46	238	67	33	18	0.078 (±0.1033)	0.075 (±0.0962)	0.035 (±0.0212)
MYK	11	238	58	13	4	0.273 (±0.1618)	0.254 (±0.1430)	0.057 (±0.0340)
ISG	14	238	59	23	9	0.199 (±0.2076)	0.168 (±0.1504)	0.065 (±0.0377)
TKT	11	238	78	29	4	0.241 (±0.1790)	0.273 (±0.1669)	0.102 (±0.0548)

^a^ Average (±*SD*) observed heterozygosity over polymorphic sites.

^b^ Average (±*SD*) expected heterozygosity over polymorphic sites.

^c^ Probability that two randomly chosen alleles at a homologous locus are different.

### 3.3 Phylogenetic relationship and mimetic characters of *P. polytes* in the Ryukyu Islands

**Figure 4:**
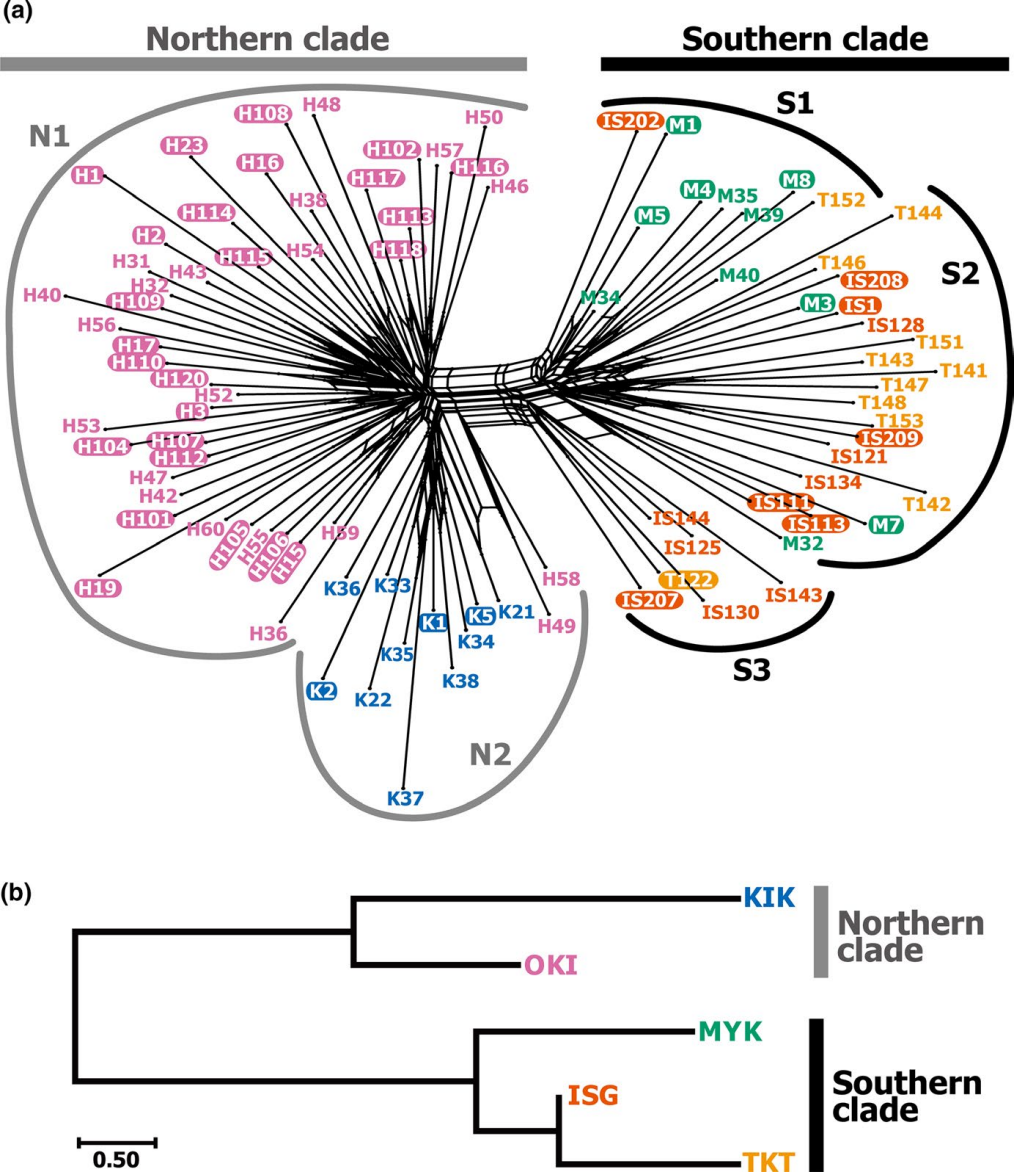
An individual‐level phylogenic network and a population‐level phylogenetic tree of *P. polytes* from the five islands. (a) Phylogenetic network estimated from concatenated SNP site data. Colors indicate the island where the sample was collected: blue, KIK; pink, OKI; green, MYK; orange, ISG; yellow, TKT. Mimetic individuals are denoted as white outlined letters in the sample names. N1–2 and S1–3 indicate subclades within northern and southern clades, respectively. (b) The population‐level tree among the five islands estimated by the neighbor‐joining method (Saitou & Nei, 1987) based on the genetic distances *D_A_* among islands calculated from concatenated SNP site data. The scale bar below the tree indicates the genetic distances in units of the net number of nucleotide differences of the SNP sites

**Figure 5:**
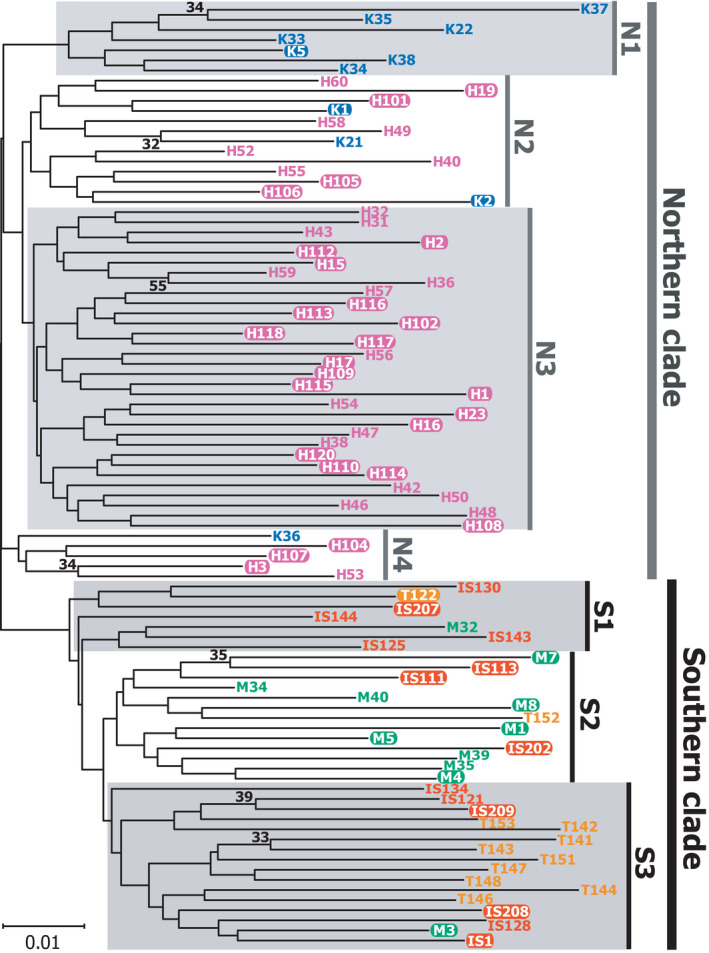
Molecular phylogenic tree of *P. polytes* from the five islands. The neighbor‐joining tree was estimated from the concatenated SNP site data. Numbers on the tree indicate support values for the node of the tree estimated from 1,000 bootstrap replications. Colors indicate the island where the sample was collected: blue, KIK; pink, OKI; green, MYK; orange, ISG; and yellow, TKT. Mimetic individuals are denoted as white outlined letters for the sample name. N1–4 and S1–3 indicate subclades within northern and southern clades, respectively, implied from this phylogenetic tree. The scale bar indicates the estimated evolutionary distances in units of the number of nucleotide substitutions per site

We again identified separate northern (KIK and OKI) and southern (MYK, ISG, and TKT) island groups by molecular phylogenetic analyses (Figures 4 and 5). A phylogenetic network based on concatenated SNP site data clearly indicated two large, northern and southern clades (Figure 4a), which contained subclades mostly corresponding to individual islands. The northern subclade N1 was comprised of OKI samples, and N2 consisted of mainly KIK individuals and also included two OKI individuals. The southern subclades S1, S2, and S3 were mostly composed of MYK, TKT and ISG, and ISG samples, respectively, and were partly a mixture of other southern island individuals. The molecular phylogenetic tree (Figure 5) showed a similar subclade structure largely corresponding to KIK, OKI, MYK, ISG, and ISG and TKT Island individuals, although the separation was somewhat obscure compared with that of the phylogenetic network (Figure 4a). These two phylogenetic analyses consistently indicated a clear separation of northern and southern populations, and the mimetic phenotypes (shown by white outlined letters in Figures 4a and 5) were independent from the phylogeny, not forming monophyletic groups.

**Figure 6:**
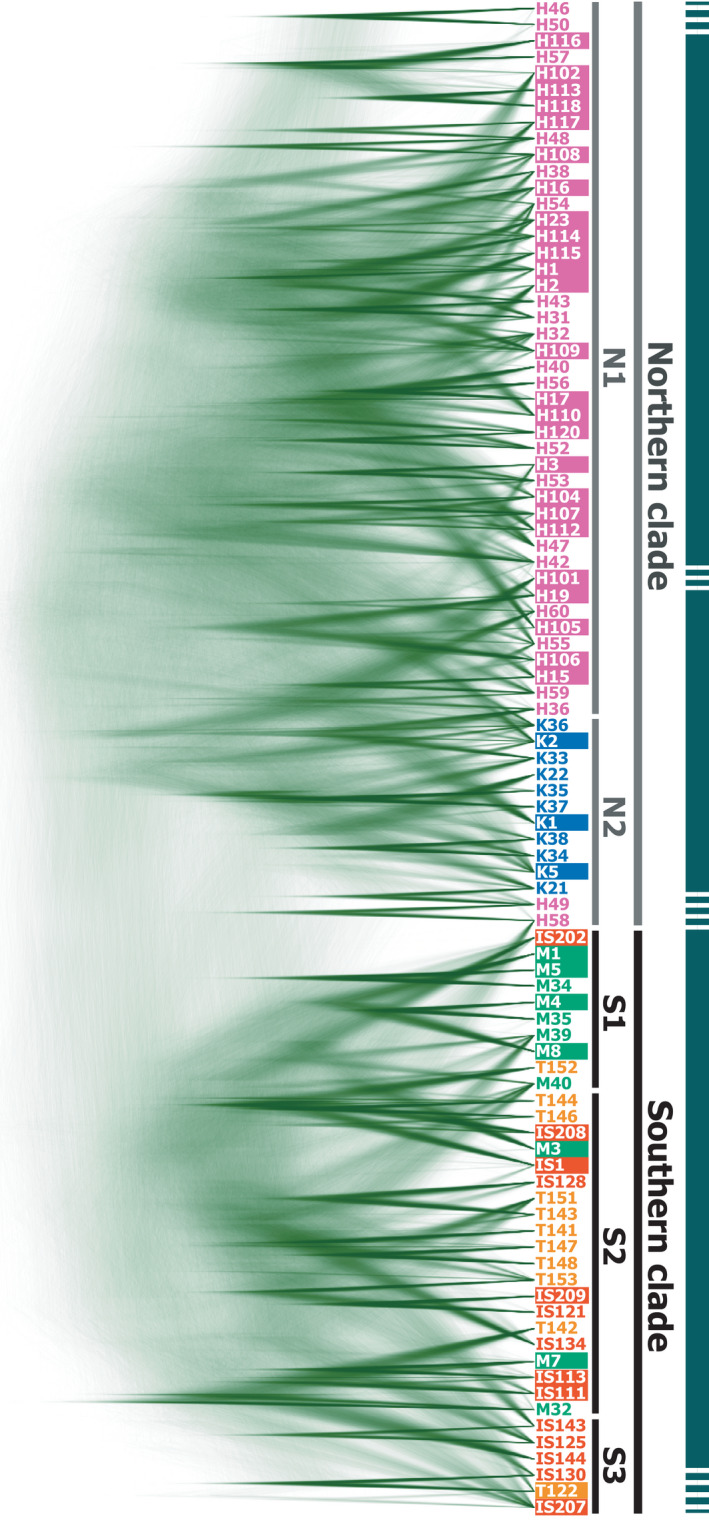
Probability density of possible coalescent trees among *P. polytes* individuals from the five islands. Colors indicate the island where the sample was collected: blue, KIK; pink, OKI; green, MYK; orange, ISG; and yellow, TKT. Mimetic individuals are denoted as white outlined letters in the sample names. Samples were ordered according to those of the phylogenetic network shown in Figure 4. N1–2 and S1–3 indicate subclades implied from the phylogenetic network and indicated in Figure 4. Green thick bar shows the clade structure implied from this coalescent‐tree probability density

**Figure 7:**
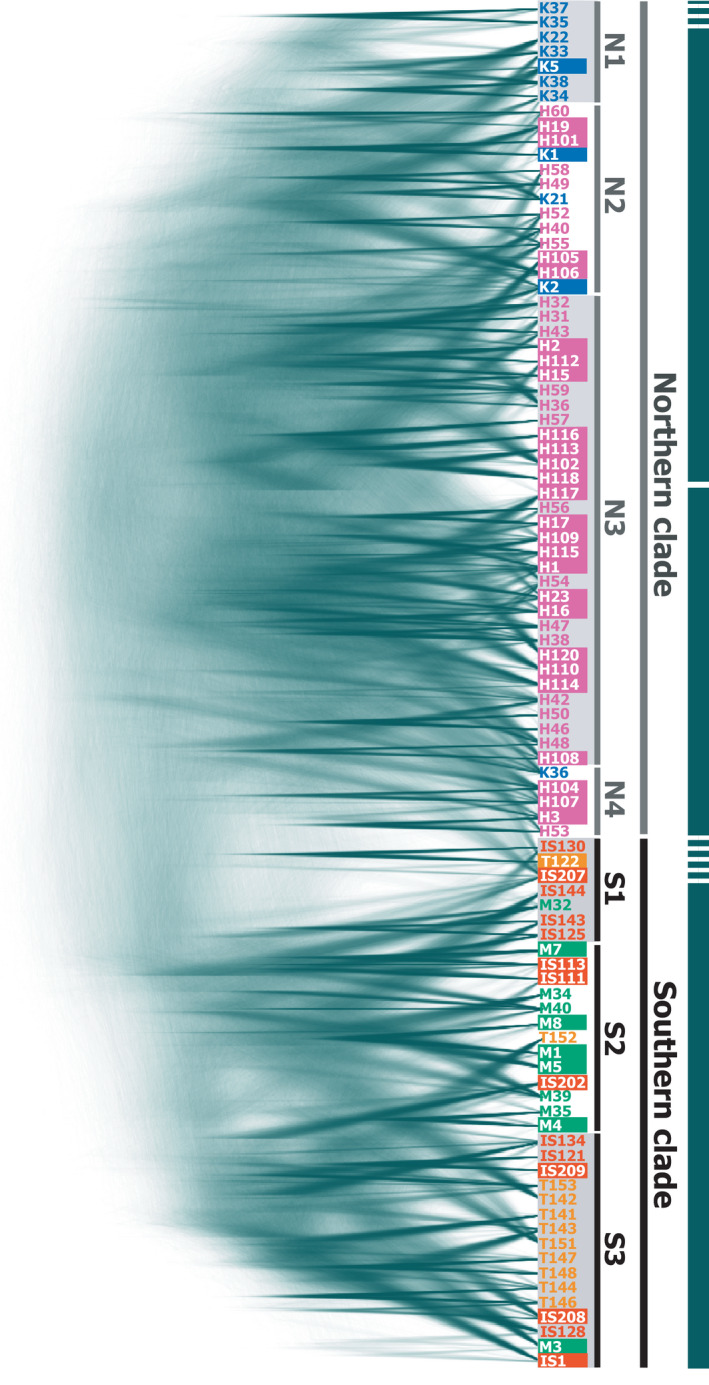
Probability density of possible coalescent trees among *P. polytes* individuals from the five islands. Colors indicate the island where the sample was collected: blue, KIK; pink, OKI; green, MYK; orange, ISG; and yellow, TKT. Mimetic individuals are denoted as white outlined letters for the sample name. Samples were ordered according to those of the neighbor‐joining phylogenetic tree shown in Figure 5. N1–4 and S1–3 indicate subclades implied from the phylogenetic tree and indicated in Figure 5. Green thick bar shows the clade structure implied from this coalescent‐tree probability density

A population‐level phylogenetic tree (Figure 4b) based on the genetic distance *D_A_* indicated a relationship among island populations, which was congruent with individual‐level phylogenetic relationships (Figures 4a and 5). The KIK and OKI populations constituted the northern clade, and MYK, ISG, and TKT constituted the southern clade, with MYK in a relatively basal position. This relationship among the island populations appeared to be consistent with those indicated by the standardized pairwise genetic differentiation scores *Φ*_ST_ of the SNP sites (Table 2). The northern KIK and OKI, and southern TKT and ISG pairs showed low (closer) scores, respectively (*Φ*_ST_, 0.125 and 0.014), and MYK showed intermediate scores relatively closer to other southern ones (*Φ*_ST_, 0.045–0.102). The island‐level branching patterns were, however, not so clear in the Slatkin's pairwise *F*_ST_ (Table 2) and phylogenetic trees (Figures 4a and 5). Such ambiguities regarding the relationships among island populations or subclades were also shown by the coalescent model‐based phylogenetic tree densities (Figures 6 and 7). The posterior density of simulated coalescent trees, however, also indicated the existence of large northern (KIK and OKI) and southern (MYK, ISG, and TKT) populations and the nonmonophyletic origin of the mimetic phenotype of *P. polytes* (indicated by white outlined letters in Figures 6 and 7).

**Table 2:** Pairwise *F*_ST_ and *Φ*_ST_ of SNP sites among the five island populations

	KIK	OKI	MYK	ISG
OKI	0.169**/0.125			
MYK	0.053/0.273	0.158**/0.202		
ISG	0.006/0.200	0.208**/0.164	0.021/0.045	
TKT	0.049/0.250	0.230**/0.201	0.046/0.102	−0.027/0.014

Note

Values on the left and right side of the slash denote pairwise *F*_ST_ (Slatkin, 1995) and *Φ*_ST_ (Nei, 1987) between each pair of islands, respectively.

** Significantly different at *p* < .01.

### 3.4 Genetic distances and other factors possibly associated with the MR of *P. polytes*

The genetic distance (*D_A_*) and MR difference among the five island populations of *P. polytes* exhibited no significant association by the Mantel test based on the nuclear SNP data obtained in this study (Table 3). On the other hand, we confirmed the strong correlation between the MR and AI controlling for the nuclear SNP‐based genetic distances by the partial Mantel test (Table 4), consistent with the results from the mtDNA data from our previous study (Tsurui‐Sato et al., 2019). Two methods of calculating AI (Tsurui‐Sato et al., 2019; Uesugi, 2000) were used, and both provided significant results at *p* < .01 and *p* < .05, respectively (bold faces in Table 4). In addition, we found a significant correlation between the geographic and genetic distances of these nuclear SNPs among the five islands by the Mantel test at *p* < .05 (Table 3), whereas such a correlation was not detected based on mtDNA analysis (Tsurui‐Sato et al., 2019).

**Table 3:** Mantel tests for mimic ratio differences (MRD) among the five islands

Independent variable	Dependent variable	*r* ^a^	*p*‐value	Reference
**AGD (ncSNPs)**	**MRD**	**−0.064**	**.369**	***This study***
AGD (mtDNA)	MRD	−0.345	.888	Tsurui‐Sato et al. (2019)
GD	MRD	−0.078	.373	Tsurui‐Sato et al. (2019)
**GD**	**AGD (ncSNPs)**	**0.899**	**.027** *	***This study***
GD	AGD (mtDNA)	0.264	.135	Tsurui‐Sato et al. (2019)
ED1	MRD	0.127	.257	Tsurui‐Sato et al. (2019)
ED2	MRD	0.108	.242	Tsurui‐Sato et al. (2019)

Note

Results of the present study are denoted by bold faces. Average genetic distances (AGD) based on nuclear SNP (ncSNPs) data were calculated as Nei's net number of nucleotide differences (*D*_A_) among island populations in the present study. AGD based on mitochondrial DNA (mtDNA) data, MRD, geographic distances (GD), and two types of environmental distances (ED1 and ED2) were estimated by Tsurui‐Sato et al. (2019). ED1 mainly reflects differences in averaged rainfall and wind speed, and ED2 mainly reflects those in temperatures among the five islands.

^a^ Correlation coefficients of the Mantel tests.

* Significantly correlated at *p* < .05.

**Table 4:** Partial Mantel tests between mimic ratio (MR) and advantage index (AI) controlling for other factors

Association	Controlled factor	*r* ^a^	*p‐*value	Reference
MR–AI	**AGD (ncSNPs)**	**0.925**	**<.001**	***This study***
	AGD (mtDNA)	0.898	<.001	Tsurui‐Sato et al. (2019)
	GD	0.913	<.001	Tsurui‐Sato et al. (2019)
	ED1	0.912	.001	Tsurui‐Sato et al. (2019)
	ED2	0.923	<.001	Tsurui‐Sato et al. (2019)
MR–AI [Uesugi]	**AGD (ncSNPs)**	**0.755**	**.011**	***This study***
	AGD (mtDNA)	0.701	.019	Tsurui‐Sato et al. (2019)
	GD	0.742	.011	Tsurui‐Sato et al. (2019)
	ED1	0.762	.01	Tsurui‐Sato et al. (2019)
	ED2	0.771	.008	Tsurui‐Sato et al. (2019)

Note

Results of the present study are denoted by bold faces. Average genetic distances (AGD) based on nuclear SNP (ncSNPs) data were calculated as Nei's net number of nucleotide differences (*D*_A_) among island populations in the present study. MR, AI, AI [Uesugi] (Sekimura et al., 2014; Uesugi, 2000), AGD based on mitochondrial DNA (mtDNA) data, geographic distances (GD), and two types of environmental distances (ED1 and ED2) were estimated by Tsurui‐Sato et al. (2019). ED1 mainly reflects differences in averaged rainfall and wind speed, and ED2 mainly reflects those in temperatures among the five islands.

^a^ Partial correlation coefficients controlled for the relevant factor.

## 4 DISCUSSION

We investigated the population genetic and phylogenetic characteristics of *P. polytes* in five islands of the Ryukyus, Japan, based on information from hundreds of nuclear SNPs (Figures 1 and 4, 5, 6, 7, Tables 1 and 2). Our results support the possibility that the MR of these butterfly populations can be explained by local model species abundance (indicated by AI or AI [Uesugi]; Table 4) rather than genetic distances or other factors such as geographic and environmental distances (Table 3). Interestingly, the current analysis based on nuclear SNPs detected a significant correlation between geographic and genetic distances (Table 3), while such a correlation was not detected by mtDNA analysis in our previous study (Tsurui‐Sato et al., 2019). This implies that the results of mtDNA analysis of one locus may have been affected by incomplete lineage sorting or smaller effective population sizes, leading to less accurate results. Hundreds of nuclear SNPs appear to reflect the microevolutionary history of *P. polytes* in the Ryukyu Islands more accurately, although SNP analysis also has inherent problems such as ascertainment bias (Hartl & Clark, 2007; Lachance & Tishkoff, 2013). This is a methodological limitation as SNP analysis often depends on common polymorphic nucleotide sites that do not necessarily represent the whole genomic and meta‐population average. In addition, the current analysis used 238 SNP sites, which is a relatively small number of genetic markers, due to our own technical limitations associated with the amount and quality of DNA samples (see Materials and Methods). The estimated genetic distances based on nuclear SNPs, however, seem to be vastly improved because they correlate with the geographic distances as expected (Table 3). These nuclear SNP‐based genetic distances, along with the geographic and environmental distances, do not explain the MR distribution across the Ryukyu Islands (Table 3). Accordingly, given that the MR and AI are strongly correlated after controlling for genetic and other distances (Table 4), we suggest that the polymorphic Batesian mimicry of *P. polytes* in the Ryukyu Islands (Figure 1) has been shaped and maintained through NFDS. This view, which has been proposed theoretically (Barrett, 1976; Kunte, 2009; Turner, 1978) and evidenced by field survey and mtDNA analysis (Tsurui‐Sato et al., 2019), was evaluated and supported by the present study by adding nuclear genomic data.

The current SNP analysis also clearly revealed the population genetic and phylogeographic characteristics of *P. polytes* in the Ryukyu Islands (Figures 1 and 4, 5, 6, 7). The results of population assignment (Figure 1b,c), nMDS (Figure 1d), and molecular phylogenetic analyses (Figures 4, 5, 6, 7) consistently segregated the two major northern and southern groups corresponding to the geographic distribution of habitat islands, not the mimetic types. Consequently, it appears that the mimetic phenotypes of female *P. polytes* are not determined by phylogenetic inertia, but exist as polymorphisms in each population, as previously indicated based on mtDNA analysis (Tsurui‐Sato et al., 2019). Mitochondrial DNA analysis also identified the large northern and southern groups of *P. polytes* in the Ryukyus, but the MYK (Miyako Island) population was clustered with the northern group (Tsurui‐Sato et al., 2019). The current analysis indicates the MYK population belongs to the southern group together with ISG and TKT (Figures 1 and 4, Table 2). This revised grouping is consistent with the known biogeographic gap, the “Kerama Strait,” dividing the fauna and flora of the Ryukyu archipelago (Figure 1; Hirao et al., 2015; Nakamura et al., 2009; Ota, 1998). The clustering of MYK with the northern group by mtDNA analysis may have been affected by incomplete lineage sorting or other factors.

The resultant view of the genetic diversity of *P. polytes* in the Ryukyus is, however, highly comparable between the previous mtDNA and current nuclear SNP analyses. We found that the southern populations (ISG and TKT) were the source of mtDNA variation, and the northern populations (KIK, OKI, and MYK) were the derived, relatively recent immigrants (Tsurui‐Sato et al., 2019) according to molecular phylogenetic, mismatch distribution, and Tajima's *D* analyses (Tajima, 1989a, 1989b). The current SNP analysis consistently indicated higher average gene diversity of SNP sites in the southern populations (0.065 and 0.102 in ISG and TKT, respectively; Table 1) and their relatively deep‐branching pattern in phylogenetic trees (Figures 4 and 5), implying higher genetic diversity and a longer history. It also indicates the relatively lower average gene diversity in the northern populations (0.048 and 0.035 in KIK and OKI, respectively; Table 1). In particular, the higher number of private alleles and the lower heterozygosity in the OKI population denote that this population harbors many low‐frequency alleles generated by relatively recent mutations (Table 1), implying closer kinships among individuals and a shorter history. These observations support the view that the northern OKI population was established by relatively recent immigrants, and some of the OKI individuals further emigrated into the northernmost KIK Island analyzed in this study (Figures 1 and 4; Tsurui‐Sato et al., 2019). The KIK population was estimated to have been established in the relatively recent past (31,000–45,000 years ago) after population expansion, based on the mtDNA variations (Tsurui‐Sato et al., 2019), which does not conflict with the relatively young age of the KIK Island that is estimated to have arisen approximately 0.85 million years ago (Osozawa et al., 2012). A migration from the south in a northerly direction also seems probable because a low‐level jet stream exists in this Ryukyu area during spring and summer (Japan Meteorological Agency, 2016). Such a passive migration due to southern wind patterns has been reported in other flying insects such as the rice planthoppers *Sogatella furcifera* and *Nilaparvata lugens* (Kishimoto, 1976; Seino et al., 1987). This phenomenon may have also affected the habitats and population genetic structure of *P. polytes*.

In summary, the findings of Tsurui‐Sato et al. (2019) and the present study suggest that the population genetic and phylogenetic characteristics of *P. polytes* in the Ryukyu Islands are highly compatible with the geographic pattern of their habitat islands, including possible migration in a northerly direction. The MR of *P. polytes* populations in each island is, on the other hand, not explained by isolation by distance or other neutral factors (Table 3), as discussed above. This suggests that, in spite of past migration and gene flow that correlates with geographic distances (Figures 1 and 4, Table 3), the MR of *P. polytes* has been adjusted to the ecological conditions of each island, as determined directly by the abundance of mimetic model species (Table 4) and indirectly by predation pressure (Katoh et al., 2017). In general, the predation pressure on prey species (*P. polytes* in this case) is difficult to evidence in wild environments. In the case of butterflies, however, the strength of predation pressure can be estimated from a field survey of beak marks (e.g., Ohsaki, 1995). Our preliminary investigation for beak marks on mimetic females of *P. polytes* in the Ryukyu Islands suggested the predation by birds, the counts of which seem to be associated with the abundance of the model species (data not shown). We propose that the strong predation pressure on this butterfly (Katoh et al., 2017), rather than isolation by distance, phylogenetic constraint, or other environmental factors, has shaped and maintained the Batesian mimicry patterns of *P. polytes* observed in the Ryukyu Islands.

Further studies are needed to explore more comprehensively the population genomic aspects of *P. polytes* in the Ryukyu Islands and molecular details and dynamics of the negative frequency‐dependent evolution of the Batesian mimicry polymorphism across the islands. The PCR‐based SNP analysis conducted herein has technical and theoretical limitations related to the number of available loci (Table 1). These may partly be caused by the relatively poor amount and quality of total DNA in a part of field samples (Table S1), leading to low‐coverage SNP loci and/or missing SNPs data, and potentially skewed results in part. An unexpected contamination from unknown parasitic/symbiotic organisms, and inherent analytical biases previously discussed (Hartl & Clark, 2007; Lachance & Tishkoff, 2013) should also be cautioned. Population‐level whole‐genome resequencing should be performed to obtain much more SNP loci from more individuals for unbiased population genomic insights about *P. polytes*. This would also enable us to address the evolution of the responsible gene, *dsx,* of polymorphic Batesian mimicry in *Papilio* butterflies (Iijima et al., 2018, 2019; Komata et al., 2016; Kunte et al., 2014; Nishikawa et al., 2015; Palmer & Kronforst, 2020; Zhang et al., 2017), which should be the focus of further investigation. This *dsx* gene may not only control the mimetic forms, but also have pleiotropic, slightly deleterious epistatic effects potentially related to the “cost of mimics” (Katoh et al., 2020). Unveiling the molecular evolutionary dynamics of the *dsx* across the Ryukyu Islands in association with the NFDS for mimetic types is necessary to understand the establishment of *P. polytes* mimicry interacting with ecological factors.

## CONFLICT OF INTEREST

None declared.

## AUTHOR CONTRIBUTIONS

**Yukuto Sato:** Conceptualization (lead); data curation (lead); formal analysis (lead); funding acquisition (equal); investigation (lead); project administration (equal); resources (lead); software (lead); supervision (equal); validation (equal); visualization (lead); writing–original draft (lead); writing–review and editing (equal). **Kaori Tsurui‐Sato:** Conceptualization (equal); data curation (equal); formal analysis (supporting); funding acquisition (supporting); investigation (equal); methodology (equal); project administration (equal); resources (supporting); software (supporting); supervision (equal); validation (equal); visualization (equal); writing–original draft (equal); writing–review and editing (esqual). **Mitsuho Katoh:** Conceptualization (supporting); data curation (equal); formal analysis (supporting); funding acquisition (equal); investigation (equal); methodology (equal); project administration (supporting); resources (equal); software (supporting); supervision (supporting); validation (equal); visualization (supporting); writing–original draft (supporting); writing–review and editing (supporting). **Ryosuke Kimura:** Conceptualization (supporting); data curation (equal); formal analysis (equal); funding acquisition (lead); investigation (supporting); methodology (equal); project administration (lead); resources (lead); software (supporting); supervision (lead); validation (lead); visualization (supporting); writing–original draft (equal); writing–review and editing (equal). **Haruki Tatsuta:** Conceptualization (equal); data curation (equal); formal analysis (equal); funding acquisition (equal); investigation (equal); methodology (lead); project administration (lead); resources (lead); software (lead); supervision (lead); validation (lead); visualization (supporting); writing–original draft (supporting); writing–review and editing (lead). **Kazuki Tsuji:** Conceptualization (lead); data curation (supporting); formal analysis (supporting); funding acquisition (lead); investigation (equal); methodology (supporting); project administration (lead); resources (lead); software (supporting); supervision (lead); validation (equal); visualization (supporting); writing–original draft (supporting); writing–review and editing (equal).

## Supporting information

Table S1

## Data Availability

Sequencing reads are available at the DDBJ Sequence Read Archive (DRA) under the accession number DRA010473. The data set and scripts of the Mantel and partial Mantel tests were provided at the Dryad, an international open‐access repository of research data, with the doi (digital object identifier) number of https://doi.org/10.5061/dryad.p8cz8w9ns.
