# Supplementary material for: Population genetic structure and evolution of Batesian mimicry in Papilio polytes from the Ryukyu Islands, Japan, analyzed by genotyping‐by‐sequencing
Source: Ecol Evol. 2020 Dec 24;11(2):872–86. doi: 10.1002/ece3.7092 (PMC7820160; doi:10.1002/ece3.7092)
Supplement: Supplementary file 1 — Table S1 [file ECE3-11-872-s001.docx]

| **Table S1. Total DNA concentration and quality of *Papilio polytes* samples of the present study.** | | | |
| --- | --- | --- | --- |
| Sample name | Location (shown in Fig. 1) | DNA concentration (ng/ul) | OD_260_/OD_280_ |
| K1 | KIK | 7.1 | 1.05 |
| K2 | KIK | 12.3 | 0.93 |
| K21 | KIK | 20.6 | 1.29 |
| K22 | KIK | 17.7 | 2.51 |
| K33 | KIK | 7.7 | 1.24 |
| K34 | KIK | 10.9 | 1.47 |
| K35 | KIK | 9.6 | 3.38 |
| K36 | KIK | 19.5 | 1.83 |
| K37 | KIK | 10.2 | 2.39 |
| K38 | KIK | 9.9 | 1.33 |
| K5 | KIK | 4.4 | 2.21 |
| H1 | OKI | 20.3 | 1.85 |
| H101 | OKI | 15.5 | 1.87 |
| H102 | OKI | 15.5 | 1.96 |
| H104 | OKI | 22.9 | 2.75 |
| H105 | OKI | 19.6 | 1.97 |
| H106 | OKI | 25.8 | 1.22 |
| H107 | OKI | 14.0 | 2.09 |
| H108 | OKI | 19.2 | 2.51 |
| H109 | OKI | 26.2 | 1.62 |
| H110 | OKI | 15.2 | 2.89 |
| H112 | OKI | 23.2 | 2.58 |
| H113 | OKI | 16.6 | 1.49 |
| H114 | OKI | 11.7 | 1.70 |
| H115 | OKI | 13.3 | 1.86 |
| H116 | OKI | 17.9 | 1.87 |
| H117 | OKI | 16.2 | 1.41 |
| H118 | OKI | 17.9 | 2.51 |
| H120 | OKI | 7.9 | 1.63 |
| H15 | OKI | 29.7 | 2.26 |
| H16 | OKI | 11.7 | 0.99 |
| H17 | OKI | 11.1 | 0.98 |
| H19 | OKI | 17.2 | 1.97 |
| H2 | OKI | 8.4 | 1.17 |
| H23 | OKI | 24.1 | 1.67 |
| H3 | OKI | 21.8 | 2.20 |
| H31 | OKI | 14.9 | 1.91 |
| H32 | OKI | 44.3 | 2.55 |
| H36 | OKI | 23.0 | 2.41 |
| H38 | OKI | 26.4 | 2.37 |
| H40 | OKI | 28.8 | 2.21 |
| H42 | OKI | 9.5 | 1.91 |
| H43 | OKI | 9.1 | 1.67 |
| H46 | OKI | 12.6 | 2.28 |
| H47 | OKI | 21.6 | 3.32 |
| H48 | OKI | 15.4 | 1.37 |
| H49 | OKI | 9.2 | 3.78 |
| H50 | OKI | 12.0 | 2.08 |
| H52 | OKI | 15.3 | 1.86 |
| H53 | OKI | 23.9 | 2.02 |
| H54 | OKI | 17.6 | 1.73 |
| H55 | OKI | 16.7 | 2.45 |
| H56 | OKI | 14.1 | 1.01 |
| H57 | OKI | 25.6 | 1.59 |
| H58 | OKI | 6.1 | 0.89 |
| H59 | OKI | 25.6 | 1.88 |
| H60 | OKI | 16.8 | 2.60 |
| M1 | MYK | 8.9 | 1.31 |
| M3 | MYK | 6.7 | 2.44 |
| M9 | MYK | 18.2 | 2.21 |
| M32 | MYK | 10.3 | 2.20 |
| M34 | MYK | 1.6 | 0.54 |
| M35 | MYK | 12.8 | 2.22 |
| M39 | MYK | 5.8 | 2.56 |
| M4 | MYK | 6.2 | 0.91 |
| M40 | MYK | 7.8 | 1.94 |
| M5 | MYK | 9.4 | 0.85 |
| M7 | MYK | 11.8 | 2.18 |
| M8 | MYK | 10.2 | 4.42 |
| IS1 | ISG | 29.0 | 2.02 |
| IS111 | ISG | 11.1 | 1.34 |
| IS113 | ISG | 13.9 | 2.39 |
| IS121 | ISG | 9.9 | 1.32 |
| IS125 | ISG | 15.1 | 2.15 |
| IS128 | ISG | 14.2 | 2.62 |
| IS130 | ISG | 16.0 | 2.05 |
| IS134 | ISG | 14.4 | 2.07 |
| IS143 | ISG | 10.0 | 2.01 |
| IS144 | ISG | 16.7 | 2.08 |
| IS202 | ISG | 18.4 | 1.85 |
| IS207 | ISG | 13.3 | 2.06 |
| IS208 | ISG | 10.5 | 1.45 |
| IS209 | ISG | 14.5 | 1.47 |
| T121 | TKT | 15.5 | 2.23 |
| T122 | TKT | 17.8 | 2.20 |
| T141 | TKT | 9.7 | 6.50 |
| T142 | TKT | 18.5 | 2.14 |
| T143 | TKT | 8.8 | 2.22 |
| T144 | TKT | 12.4 | 1.15 |
| T146 | TKT | 12.4 | 1.28 |
| T147 | TKT | 16.1 | 3.38 |
| T148 | TKT | 18.8 | 2.08 |
| T151 | TKT | 15.6 | 3.19 |
| T152 | TKT | 19.5 | 2.22 |
| T153 | TKT | 23.7 | 2.57 |
